# Resting state network mapping in individuals using deep learning

**DOI:** 10.3389/fneur.2022.1055437

**Published:** 2023-01-12

**Authors:** Patrick H. Luckett, John J. Lee, Ki Yun Park, Ryan V. Raut, Karin L. Meeker, Evan M. Gordon, Abraham Z. Snyder, Beau M. Ances, Eric C. Leuthardt, Joshua S. Shimony

**Affiliations:** ^1^Division of Neurotechnology, Department of Neurological Surgery, Washington University School of Medicine, St. Louis, MO, United States; ^2^Mallinckrodt Institute of Radiology, Washington University School of Medicine, St. Louis, MO, United States; ^3^Department of Physiology and Biophysics, University of Washington, Seattle, WA, United States; ^4^MindScope Program, Allen Institute, Seattle, WA, United States; ^5^Department of Neurology, Washington University School of Medicine, St. Louis, MO, United States; ^6^Department of Neuroscience, Washington University School of Medicine, St. Louis, MO, United States; ^7^Department of Biomedical Engineering, Washington University in St. Louis, St. Louis, MO, United States; ^8^Department of Mechanical Engineering and Materials Science, Washington University in St. Louis, St. Louis, MO, United States; ^9^Center for Innovation in Neuroscience and Technology, Division of Neurotechnology, Washington University School of Medicine, St. Louis, MO, United States; ^10^Brain Laser Center, Washington University School of Medicine, St. Louis, MO, United States; ^11^National Center for Adaptive Neurotechnologies, Albany, NY, United States

**Keywords:** deep learning, machine learning, resting state functional MRI, representation of function, brain mapping

## Abstract

**Introduction:**

Resting state functional MRI (RS-fMRI) is currently used in numerous clinical and research settings. The localization of resting state networks (RSNs) has been utilized in applications ranging from group analysis of neurodegenerative diseases to individual network mapping for pre-surgical planning of tumor resections. Reproducibility of these results has been shown to require a substantial amount of high-quality data, which is not often available in clinical or research settings.

**Methods:**

In this work, we report voxelwise mapping of a standard set of RSNs using a novel deep 3D convolutional neural network (3DCNN). The 3DCNN was trained on publicly available functional MRI data acquired in *n* = 2010 healthy participants. After training, maps that represent the probability of a voxel belonging to a particular RSN were generated for each participant, and then used to calculate mean and standard deviation (STD) probability maps, which are made publicly available. Further, we compared our results to previously published resting state and task-based functional mappings.

**Results:**

Our results indicate this method can be applied in individual subjects and is highly resistant to both noisy data and fewer RS-fMRI time points than are typically acquired. Further, our results show core regions within each network that exhibit high average probability and low STD.

**Discussion:**

The 3DCNN algorithm can generate individual RSN localization maps, which are necessary for clinical applications. The similarity between 3DCNN mapping results and task-based fMRI responses supports the association of specific functional tasks with RSNs.

## 1. Introduction

It is well known that intrinsic neural activity is temporally correlated within widely distributed brain regions that simultaneously respond to imposed tasks (1). This phenomenon is known as resting functional connectivity, and the associated topographies are known as resting state networks (RSNs) or intrinsic connectivity networks (2, 3). Resting state functional connectivity can be studied using both invasive and non-invasive electrophysiology (4, 5). However, the majority of research on resting state functional connectivity focuses on blood oxygen level dependent (BOLD) functional magnetic resonance imaging (fMRI), which, in the absence of specific tasks (e.g., finger tapping), is referred to as resting state fMRI (RS-fMRI). Correlation analysis of these fluctuations identifies spatial patterns of functional connectivity widely known as RSNs (6, 7).

RS-fMRI studies have yielded a better understanding of normal brain functional organization and the pathological changes that occur in neuropsychiatric disorders, e.g., Alzheimer's disease, HIV infection, autism, Parkinson's disease, Down syndrome, and others (8–14). RS-fMRI mapping also has applications in pre-surgical planning of operative procedures for the treatment of brain tumors and repetitive trans-cranial magnetic stimulation for depression (15–20). However, reliable results often need a large amount of data which can be difficult to acquire in some patient populations (21, 22).

Deep learning (DL) is a branch of machine learning that has become widely used in multiple domains. DL is a form of artificial neural networks comprising multiple “hidden” layers between the input and output, which simultaneously perform feature selection and input/output mapping by adjusting network weights during training. DL models have achieved state-of-the-art performance on numerous tasks, often times comparable to or exceeding human performance (23–25). This development has led to the adoption of DL in medical research, with the ultimate goal of achieving precision medicine at the individual patient level (26–29). Applications of deep learning to neuroimaging data range from artifact removal, normalization/harmonization, quality enhancement, and lowering radiation/contrast dose (30–36).

Defining RSNs accurately is important and difficult and there is no established method to do so. Thus, in this study we trained a deep three-dimensional convolutional neural network (3DCNN) utilizing a large cohort of healthy participants (*n* = 2,010) across a wide age range to generate maps that represent the probability membership of a voxel belonging to a particular RSN. These maps are referred to herein as voxelwise RSN membership probability maps. Model results were compared to publicly available RS-fMRI (37) and aggregated task fMRI (T-fMRI) mappings compiled in the Neurosynth platform (www.neurosynth.org) (38). The trained model was further evaluated for stability given varying quantities of resting state fMRI data and levels of added noise. Voxelwise RSN membership probability maps (group mean and standard deviation) were derived using the model results from all available data and are made publicly available. The 3DCNN algorithm can generate individual RSN localization maps, which are necessary for clinical applications (17, 39).

## 2. Materials and methods

### 2.1. Data

Normal human RS-fMRI data (*N* = 2,010) were obtained from the control arms of primary studies designed, conducted, and analyzed external to the derivative study described herein. These include the Brain Genomics Superstruct Project (40) (GSP) and ongoing studies at Washington University in St. Louis, including healthy control data from the Alzheimer's Disease Research Center and Neurodegeneration studies (Table 1). All participants were cognitively normal based on study-specific performance testing. The appropriate Institutional Review Board approved all studies, and all participants provided written informed consent for the given study, which also allowed de-identified data for use in this derivative study.

**Table 1:** Characteristics of training data.

	**Total**	**HIV**	**ADRC**	**GSP**
Number of participants	2,010	206	665	1,139
Mean and STD of age	44.6 ± 23.5	37.9 ± 17.1	67.6 ± 7.8	21.3 ± 2.7
% Female	59%	52%	60%	59%
Mean and STD for education	14.8 ± 2.2	13.9 ± 2.1	15.9 ± 2.6	14.3 ± 1.9
% Caucasian	69%	44%	86%	65%
Scanner		Trio/Prisma	Trio/Biograph	Trio
Voxel Size (mm^3^)		4	4	3
Repetition time (ms)		2,200	2,200	3,000
Time to Echo (ms)		27	27	30
Flip angle (degrees)		90	90	85

### 2.2. Magnetic resonance imaging (MRI) acquisition

All neuroimaging was performed on 3T Siemens scanners (Siemens AG, Erlangen, Germany) equipped with the standard 12-channel head coil (Table 1). A high-resolution, 3-dimensional, sagittal, T1-weighted, magnetization-prepared rapid gradient echo scan (MPRAGE) (echo time [TE] = 1.54–16 ms, repetition time [TR] = 2200–2,400 ms, inversion time = 1,000–1,100 ms, flip angle = 7–8°, 256 × 256 acquisition matrix, 1.0–1.2 mm^3^ voxels) and T2-weighted fast spin echo sequence (FSE) (TR = 3,200 ms, TE = 455 ms, 256 x 256 acquisition matrix, 1 mm isotropic voxels) were acquired. RS-fMRI scans were collected using a gradient spin-echo sequence (voxel size = 3–4 mm^3^, TR = 2,200–3,000 ms, FA = 80–90°) sensitive to BOLD contrast. Statistical analysis of network functional connectivity (evaluated within the default mode and dorsal attention network) between the different data sets revealed no major group differences (Supplementary Figure 1). Each participant contributed ~7–14 min of resting state fMRI data, processed using standard methods developed at Washington University (41). Table 1 provides study-specific details related to RS-fMRI image acquisition.

### 2.3. MRI processing

RS-fMRI data were preprocessed using previously described techniques including non-linear atlas registration (42). Dynamic fMRI data was adjusted to obtain consistent imaging intensities across slices, thereby accounting for interleaved acquisitions and differential timings across slices. fMRI data was also adjusted for head motion within scan sessions and across scan sessions for each subject using rigid-body transformations. We further censored fMRI time frames by excluding frames exceeding 0.5% of the root-mean-squared intensity variation of scanned frames (41, 43). All fMRI data were affinely transformed to a standardized atlas generated from T1-weighted images historically acquired from healthy, young adults on Siemens Trio scanners at Washington University (https://4dfp.readthedocs.io/en/latest). Whenever available (HIV and ADRC), affine transformations used T2-weighted images for refinement of spatial normalization. Typical composite transformations provided affine mappings of fMRI to T2-weighted images to MPRAGE to the standardized atlas. Spatial normalization also involved exclusion of transient magnetization precessions in the initial five frames of fMRI, Gaussian filtering with isotropic kernels of 6 mm full-width at half-maximum, removal of linear fMRI signal trends within scan sessions, low pass filtering at 0.1 Hz, removal of linear regressions for head motion, removal of fMRI time series localized to white matter or CSF, and removal of fMRI time series averaged over the whole brain. The latter global signal regression ensured that subsequent calculations of correlation-related measures were zero-centered partial correlations controlling for brain-wide variances (44). Volume-dependent nuisance regressors derived from segmentations generated by FreeSurfer for each subject (http://surfer.nmr.mgh.harvard.edu). For visualizations, all imaging was affinely transformed to high-resolution MNI atlases.

### 2.4. 3DCNN

A 3D convolutional neural network (3DCNN) with 74 layers was trained to classify each gray matter voxel to a given RSN. The 3DCNN was implemented in Matlab R2019b (www.mathworks.com). It had a densely connected architecture (45), with residual layers (46) nested within each of the three dense blocks. Within the network, 1 × 1 × 1, 3 × 3 × 3, and 7 × 7 × 7 convolutions were performed. The final output, as well as the output from each dense block was directly connected to the cross entropy layer after global average pooling and 20% dropout. This training strategy has been shown to prevent overfitting through structural regularization, is more robust to spatial translations of the input, and requires fewer learnable parameters (47, 48). Batch normalization was used prior to convolutional operations within the network. Leaky rectified linear units were used after convolutions. Both max and average pooling were used between dense blocks for dimensionality reduction. Combining max and average pooling has been shown in some studies to outperform either technique on its own (49). Each pooling layer was 2 × 2 × 2 with a stride of 2. Supplementary Figure 2 shows the 3DCNN architecture. Because the number of samples from each class (RSN) was not constant, the 3DCNN used a cross entropy loss function with weighted classification such that each class contributed equally to the loss function. Training was terminated if the accuracy did not improve after three validations.

### 2.5. Training data

Predefined seed regions of interest (50) (300 ROIs, https://greenelab.ucsd.edu/data_software) were used to assign voxels to one of 13 RSNs for generation of training data. The networks include dorsal somatomotor (SMD), lateral somatomotor (SML), cinguloopercular (CON), auditory (AUD), default mode (DMN), parietal memory (PMN), visual (VIS), frontoparietal (FPN), salience (SAL), ventral attention (VAN), dorsal attention (DAN), medial temporal (MTL), and reward (REW). Training sample connectivity maps for each RSN were generated by random sampling of voxels within ROIs of a given network. The RS-fMRI signal within these voxels was averaged and used to extract a whole brain 3D similarity map. For example, for a given network (X) and an individual scan, we generate a single training sample by subsampling ROIs known to belong to X, averaging those ROIs together, and calculating the similarity of the mean signal with the rest of the voxels in the brain. Similarity was calculated by computing both the Pearson product moment correlation and Euclidean Distance between the mean of the subsampled RS-fMRI signals and all other voxels in the brain. The 3D similarity map was then assigned to the selected RSN (after confirming that it had the highest correlation between the mean subsampled signal and the mean signal for each network). The assigned network labels were used for classification during training of the 3DCNN. This process was applied in numerous iterations for each network and for each participant. Supplementary Figure 3 shows examples of 3D similarity maps of the DMN, FPN, and SMD used for training. A total of 1,313,140 training instances (~100,000 per network) were generated across all networks. During training, samples were augmented by a combination of 3D random affine transformations [rotations (±5 degrees), translations (±3 pixels)], intensity scaling (between 0.9 and 1.1), shearing (±3Degrees), and adding gaussian noise. Data augmentation has been shown in numerous studies to improve out of sample testing and prevent overfitting (51). Two hundred fMRI scans from our training data set (approximately 10% of the data) stratified based on age, gender, and study were reserved for generating validation data for the 3DCNN, and validation samples were generated in the same manner as above. Approximately 200,000 validation samples (~15,000 per network) were generated from the held out scans.

### 2.6. Testing data

After training, model outputs were compared using RS-fMRI data from the Midnight Scan Club (39) (MSC) collected at Washington University. The MSC contains high quality data collected from 10 participants, each scanned for 30 min in 10 separate sessions. The MSC data are freely avaliable (https://www.openfmri.org/dataset/ds000224/) and have been thoroughly characterized in numerous studies (39, 52–54). MSC data were used to evaluate model performance given a reduced quantity of fMRI data, and to evaluate model performance after noise addition. Unstructured noise addition was performed by adding the scaled fMRI data to scaled pink noise. For example, to achieve 10% noise injection, the fMRI data was rescaled to a [−0.9 0.9] interval, the noise signal was scaled to a [−0.1 0.1] interval, and the signals were added together. A new noise signal was generated for each voxel. Similarity between results was measured using the multiscale structural similarity index (MSSI) (55). MSSI estimates similarity of a pair of 3D volumes by first estimating similarity of the pair for successively down-sampled versions of the pair, typically down-sampling by factors of 2, 4, 8, 16, and 32. Down-samplings are weighted according to a Gaussian distribution peaked at the middle spatial factor to mimic human visual sensitivity for spatial patterns. In the current state of the art, RSN topographies are identified, and distinguished from noise or artifacts, by human visual assessments. Whether by seed correlations, factorizations such as independent component analysis, or community detection algorithms, final adjudication involves prior expectations of RSN topographies derived from reported studies. MSSI represents one of the best performing implementations of objective structural similarity indices which posit that human visual assessments are effectively ground truths. Structural similarity provides better metrics of human visual assessments than simpler metrics such as mean squared error, peak signal-to-noise ratio, and their variations that invoke penalizations. Structural similarity indices are especially suited for assessment of RSN features because it compares imaging instances with arbitrary reference imaging providing effective ground truths (56). For visualization purposes the final outputs were mapped to an average surface space with the methods described in using “Conte69 atlas fs_LR” surface using HCP workbench (53, 57).

### 2.7. RSN characterization

Mean voxelwise RSN membership probability maps were generated by averaging the 3DCNN output for each subject in our sample for each RSN separately. The same data set was used to generate a voxelwise standard deviation (STD) map and a voxelwise map of the mean divided by the STD. These maps were then used to create RSN summary measures as the area included in each RSN, averages of the mean and mean/STD over the extent of each RSN based on the winner take all maps (see below).

To evaluate our results in the context of traditional, seed-based correlation analysis, we correlated RSN membership probabilities produced by the 3DCNN and compared those to traditional time series correlation matrices. The 3DCNN correlation matrices were generated by correlating the softmax output probabilities, while the functional connectivity matrices were generated by correlating the RS-fMRI time series at both the voxel and ROI level.

### 2.8. Comparison data

As a partial validation of our method we compared our results with two different schemes. First, we compared our results with RSN maps published by Dworetsky et al. (37). Their study generated probabilistic mappings of RSNs by using data from five independent data sets, one for data-driven network discovery and template creation, the second for template matching and probability mapping, and the final three for replication of the probabilistic maps. Second, we compared our results with T-fMRI results generated in the Neurosynth platform. The Neurosynth platform (neurosynth.org) can generate statistical maps of significance of T-fMRI responses to behavioral paradigms (38). In brief, Neurosynth parses texts of published T-fMRI studies to generate aggregrated task activation data linked to user-selected search terms, e.g., “language.” Neurosynth maintains a large database of publications on T-fMRI, which it parses for a set of pre-defined term and activations related to brain function paradigms. When an article uses a term, Neurosynth attempts to extract brain regions that were consistently reported in the tables of that study. The list of terms used in our analysis were “attention” (corresponding to DAN), “auditory,” “default mode,” “language” (corresponding to VAN), “motor” (corresponding to SMD), “reward,” and “visual.” For both comparisons, similarity was measured using the MSSI.

## 3. Results

Participant demographics are shown in Table 1. A majority of the cohort were Caucasian (69%) females (59%), with an average age of 44.6 ± 23.5 years and 14.8 ± 2.2 years of education. Supplementary Figure 4 shows the age distribution based on the studies used in the analysis.

### 3.1. Model results

**Figure 1:**
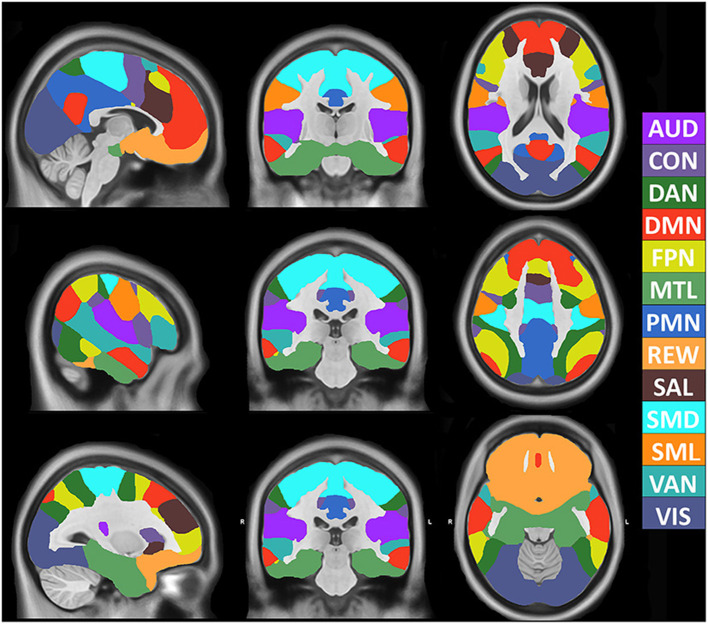
Volumetric segmentation of resting state networks based on maximum probability produced by the 3DCNN averaged across 2010 participants. SMD, dorsal somatomotor; SML, lateral somatomotor; CON, cinguloopercular; AUD, auditory; DMN, default mode; PMN, parietal memory; VIS, visual; FPN, frontoparietal; SAL, salience; VAN, ventral attention; DAN, dorsal attention; MTL, medial temporal; and REW, reward.

**Figure 2:**
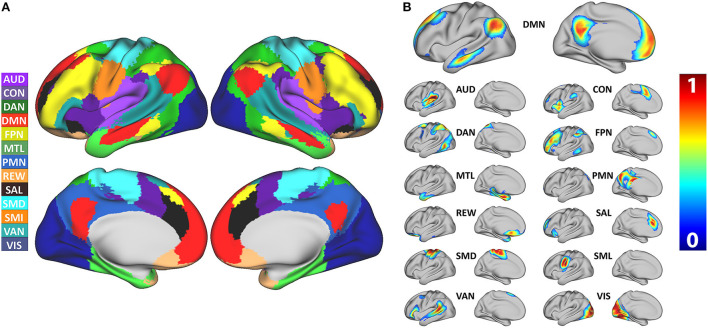
**(A)** Surface segmentation of resting state networks based on maximum probability produced by the 3DCNN averaged across 2,010 participants. **(B)** Probability maps of individual resting state networks averaged across 2,010 participants. SMD, dorsal somatomotor; SML, lateral somatomotor; CON, cinguloopercular; AUD, auditory; DMN, default mode; PMN, parietal memory; VIS, visual; FPN, frontoparietal; SAL, salience; VAN, ventral attention; DAN, dorsal attention; MTL, medial temporal; and REW, reward.

The model achieved 99% accuracy on training data and 96% accuracy on out of sample validation data after eight epochs (Supplementary Figure 5). After training, data from all 2,010 participants were processed with the 3DCNN, and mean and standard deviation maps were generated from those results. Figure 1 shows the RSN segmentation based on the winner take all (WTA) of softmax probabilities produced by the 3DCNN averaged across all 2010 participants. Similarly, Figure 2A displays the segmentation results projected onto the cortical surface, and Figure 2B shows the mean voxelwise RSN membership probability maps for each network (0.2 threshold). Supplementary Figure 6 shows the per-network voxel count based on a given probability threshold.

### 3.2. Model stability

**Figure 3:**
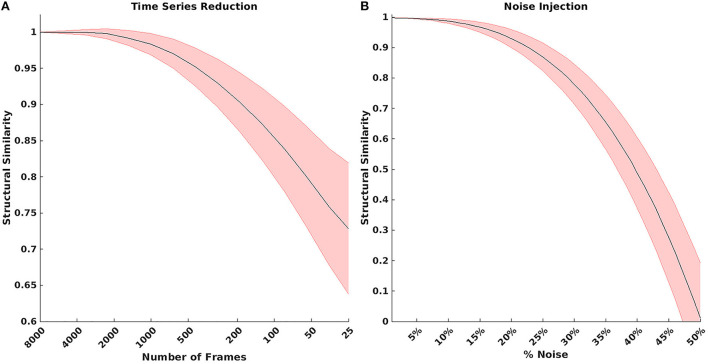
**(A)** Result of reducing the total number of BOLD time points. On average, the model maintained a 0.9 structural similarity when comparing 8,000 time points to ~150 time points. **(B)** Structural similarity when comparing model results with various amounts of noise added to the BOLD signal. The model maintained 0.9 structural similarity even after injecting 25–30% noise in the original bold signal.

The model was evaluated for stability of results based on the number of RS-fMRI time points and signal noise. Figure 3A shows the result of reducing the total number of RS-fMRI time points averaged over the MSC data. On average, the model maintained a 0.9 MSSI when comparing 8,000 time points to ~150 time points (~5:30 min). However, stability varied across networks (Supplementary Figure 7). Figure 3B shows the effect on MSSI similarity on varying levels of added pink noise. Overall, the model maintained 0.9 similarity even after the addition of 25–30% noise in the original RS-fMRI signal.

### 3.3. RSN characterization

**Figure 4:**
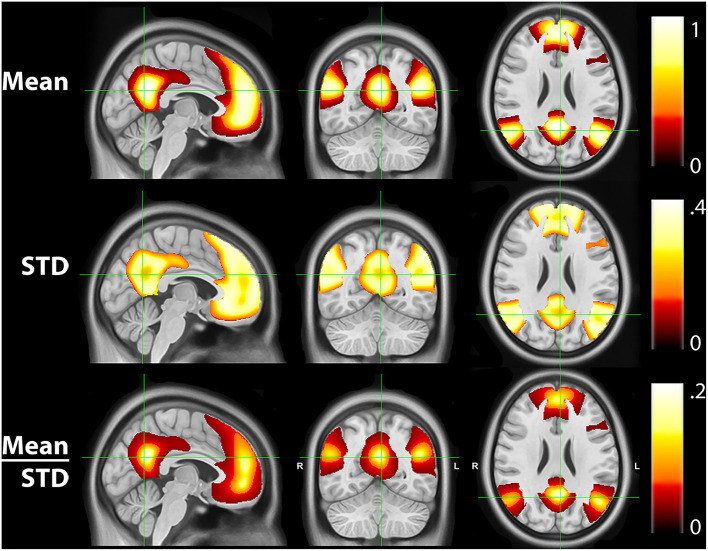
Mean, standard deviation (STD), and mean/STD probability maps for the default mode network (DMN). The mean results show a large number of voxels with high probabilities. STD maps show the majority of voxels have a relatively high STD. When scaling the mean values by the STD, the regions with high relative probabilities becomes significantly smaller.

Figure 4 shows the mean, STD, and mean/STD probability maps for the DMN (masked based on WTA probabilities from Figure 1). These results show that a large number of voxels show high mean probabilities. However, the STD maps show that the majority of those voxels have a relatively high STD, likely due to individual subject variability and limited signal to noise ratio in the data. Scaling the mean values by the STD, a measure akin to signal to noise ratio, demonstrates higher certainty of RSN membership centrally and the expected uncertainty present at the margins of the WTA regions.

**Figure 5:**
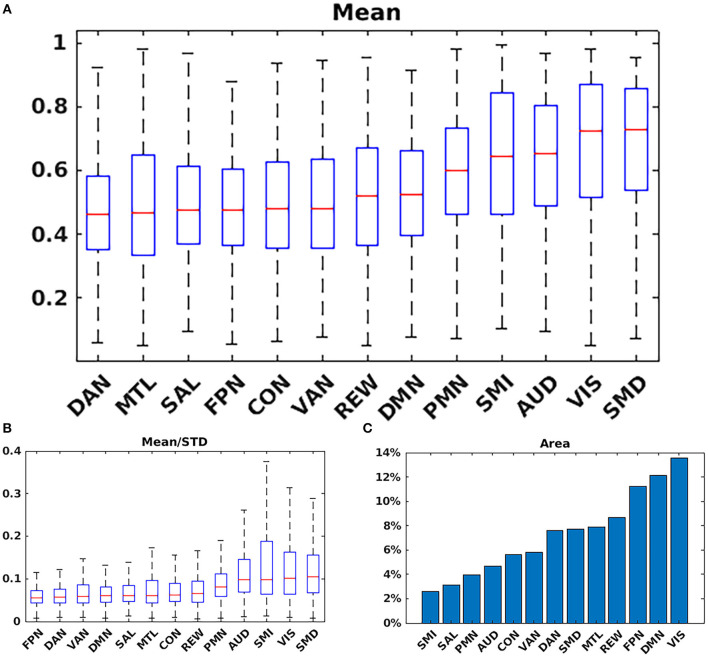
Network summary measures. **(A)** Mean probability values averaged over each RSN based on the argmax probability (Figure 1). The highest average probabilities were observed in AUD; VIS; and somatomotor networks. **(B)** Average values for the mean scaled by the standard deviation. **(C)** Total area for each network. VIS; DMN; and FPN covered the greatest area. SMD, dorsal somatomotor; SML, lateral somatomotor; CON, cinguloopercular; AUD, auditory; DMN, default mode; PMN, parietal memory; VIS, visual; FPN, frontoparietal; SAL, salience; VAN, ventral attention; DAN, dorsal attention; MTL, medial temporal; and REW, reward.

Figure 5A shows the mean probability values averaged over each RSN based on the mean WTA probability mask as shown in Figure 1. The highest average probabilities were observed in AUD, VIS, and somatomotor networks. Similarly, 5B shows the average values for the mean scaled by the standard deviation, indicating which networks have higher vs. lower individual subject variability. Lastly, 5C shows the total area for each network calculated by dividing the total number of voxels belonging to each network (Figure 1) by the total number of voxels considered in the gray matter mask. VIS, DMN, and FPN covered the greatest area.

### 3.4. Comparison with prior task and resting state and task results

**Figure 6:**
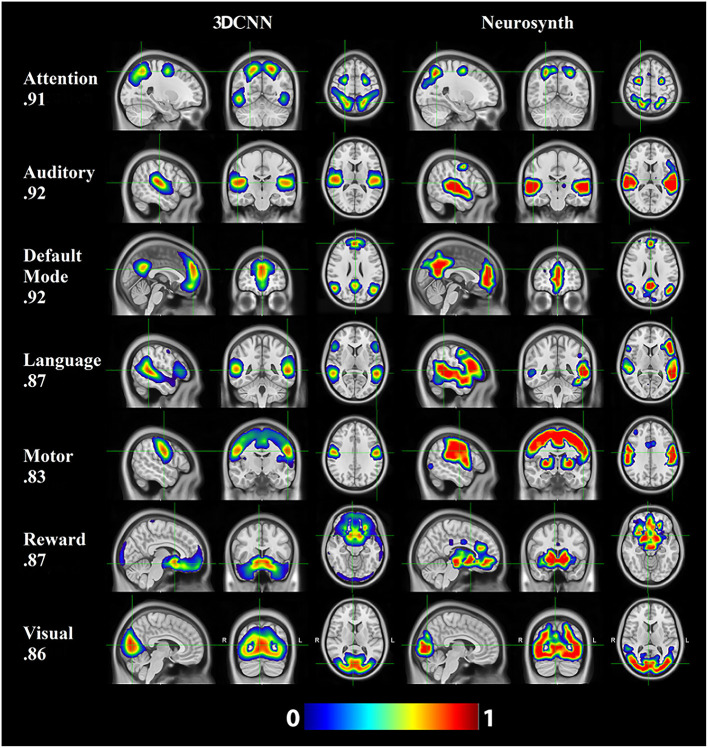
3DCNN results computed over the MSC data and functional maps generated from the Neurosynth platform. Of the networks evaluated, default mode (DMN), auditory (AUD), and attention [corresponding to dorsal attention (DAN)] showed the greatest structural similarity as measured by the MSSI.

**Figure 7:**
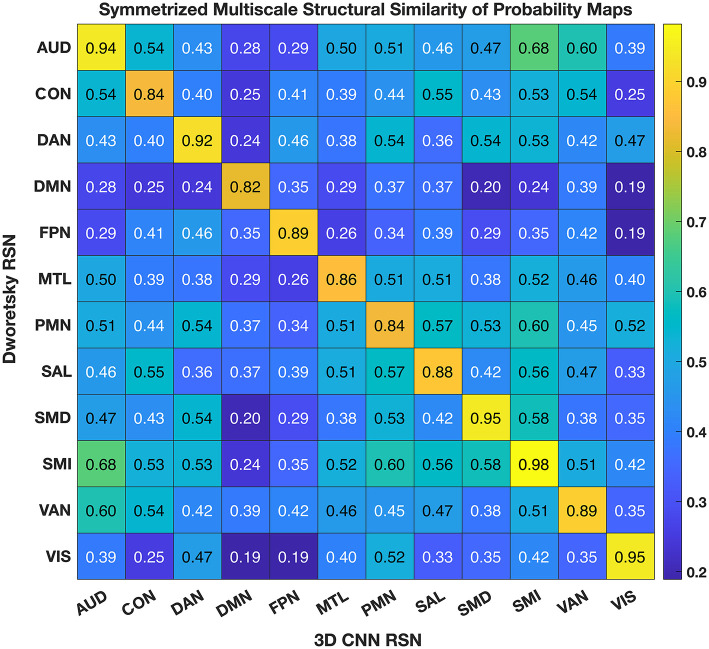
3DCNN results compared to maps published by Dworetsky et al. (37). The strongest similarities were seen in AUD; VIS; SMD; and SML. SMD, dorsal somatomotor; SML, lateral somatomotor; CON, cinguloopercular; AUD, auditory; DMN, default mode; PMN, parietal memory; VIS, visual; FPN, frontoparietal; SAL, salience; VAN, ventral attention; DAN, dorsal attention; MTL, medial temporal; and REW, reward.

Figure 6 shows the comparison of the 3DCNN results computed over the MSC data with functional maps generated from the Neurosynth platform. This comparison provides clear evidence of the similarity, at the group level, between task based functional responses and RSNs, with all spatial similarity measures being <0.83 (SMD) and peaking at 0.92 (DMN) as measured by the MSSI. Figure 7 shows the MSSI similarity comparisons to the Dworetsky et al. (37) segmentation including off-diagonal terms. The diagonal elements are all >0.8, demonstrating a high degree of overlap between RSNs determined using these two different methodologies. The strongest similarities were seen in AUD, VIS, SMD, and SML.

### 3.5. Correlation analysis

**Figure 8:**
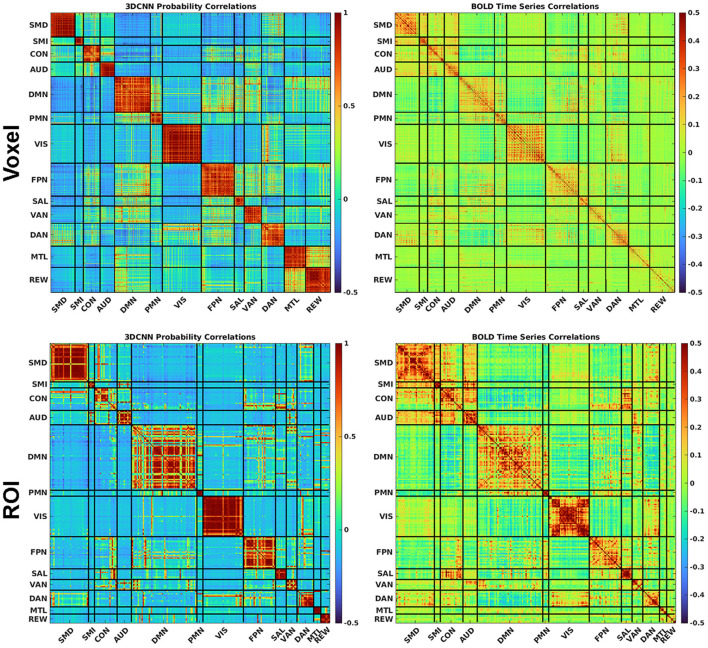
The 3DCNN allows for novel similarity measures. Softmax marginal probabilities can be correlated to generate alternative connectivity matrices between functional regions. Correlations between 3DCNN softmax probabilities are compared to correlations between BOLD time series for voxels **(top row)** and ROIs **(bottom row)**. The 3DCNN shows higher correlations and greater contrast between regions compared to conventional BOLD correlations. SMD, dorsal somatomotor; SML, lateral somatomotor; CON, cinguloopercular; AUD, auditory; DMN, default mode; PMN, parietal memory; VIS, visual; FPN, frontoparietal; SAL, salience; VAN, ventral attention; DAN, dorsal attention; MTL, medial temporal; and REW, reward.

Figure 8 shows a comparison between correlated RSN membership probabilities produced by the 3DCNN and the traditional functional connectivity matrices produced from Pearson's correlation of the time series. These results are averaged over all 2010 data samples at both the voxel level (top row) and the ROI level (bottom row). By first processing the fMRI data with the 3DCNN and then correlating softmax inferences, we see much higher correlations and greater orthogonality between regions as compared to directly correlating the fMRI time series. The greater orthogonality demonstrates the improved ability of the 3DCNN to separate between the RSN.

## 4. Discussion

Our research defines a robust voxelwise classification model of RSNs. We showed 96% validation accuracy in classifying RSNs in a large cohort of healthy participants covering a broad age range. These results were achieved on data collected from multiple scanner types and sequence parameters (Table 1). Further, the model was resilient to both noisy data and fewer time points (Figure 3). Robustness to limited and noisy data is important in clinical work and in neuroimaging studies with limited scanner time, as the reliability of functional connectivity mapping strongly depends on both the quantity and quality of available resting state data (39, 58).

The 3DCNN can be viewed as an algorithm that increases model accuracy by selecting relevant features and disregarding irrelevant, redundant, or noisy features (59). In application to RSN localization, the feature of interest is the correlation structure of multivariate data. Figure 8 shows a matrix depicting 3DCNN RSN membership probabilities in comparison to fMRI time series correlations at both the voxel and ROI levels. In this context, the 3DCNN can be viewed as a supervised feature extraction method optimized over the 13 networks with RSN membership probability as the extracted feature. The key feature in Figure 8 is the contrast between the RS-fMRI functional connectivity matrix, which shows strong, predominantly negative values in the off-diagonal blocks, vs. the result generated by the 3DCNN, which shows only minimal values in the off-diagonal blocks. The significance of this difference relates to the hierarchical structure of RSNs (60, 61). Such hierarchical organization mandates that signals generally are shared across multiple RSNs, hence, the appearance of correlations and anti-correlations in off-diagonal blocks of correlation matrices. Moreover, the total number of discrete RSNs is theoretically infinite (62) although models comprising 2, 7, and 17 RSNs exhibit particularly favorable goodness of fit criteria (63, 64). After training on a selected set of discrete RSNs, the 3DCNN assigns approximately unique RSN membership to each part of the brain. In effect the 3DCNN orthogonalizes the intrinsically hierarchical correlation structure of resting state BOLD fMRI data.

A goal of this research was to provide voxelwise group statistics based on all participants to identify ROIs with the highest network membership probabilities. Several different methods have been developed in the literature for the identification of functional connected networks using RS-fMRI data in groups and in individuals (37, 39, 65, 66). These techniques have been expanded to include sub-cortical structures, the cerebellum, and combined with other imaging modalities (67–69). Although there is no recognized gold standard for network mapping, our results are similar to results obtained using different methodologies (37, 38, 70). Our published maps contribute to this literature and, given the large sample size and model robustness, provide an advantageous set of ROIs for group level RS-fMRI analysis that could be used in future studies.

We compared our results with probabilistic maps derived from multiple RS-fMRI data-sets recently published by Dworetsky et al. (37) (Figure 7). The AUD, VIS, SMD, and SML networks show the highest correspondence between the RSN maps proposed by Dworetsky et al. (37) and the present results. The same networks show the greatest consistency across subjects (expressed in terms of SNR) in Figure 5. Thus, RSNs involving primary cortical areas exhibit the greatest consistency across individuals and datasets whereas RSNs involving “higher order” functional systems, e.g., those associated with cognitive control (CON) and memory (DMN), are topographically more variable. These observations are consistent with prior results showing that association system RSNs are more variable across individuals in comparison to RSNs representing sensory and motor functions (71). In contrast, functional connectivity within primary cortical areas is more variable within individuals and across sessions, a phenomenon most likely reflecting differences in levels of arousal (72, 73).

Resting state functional connectivity analysis began with the observation that the topography of spontaneous activity correlations within the somatomotor system replicates finger-tapping task responses (1). This result was later extended to other tasks, giving rise to the notion that RSNs can be associated with specific sensory, motor, and cognitive functions (74). However, with respect to “higher order” RSNs, these associations appear to be incompletely specific and dependent on the details of the imposed task (75, 76). Thus, the objective of associating RSNs with specific cognitive processes is far from trivial. In this context, we report a comparison of a subset of current RSNs with task-based responses aggregated in the Neurosynth platform (Figure 6). The key word used to search Neurosynth is listed at the left of each row. This comparison provides clear evidence of the similarity, at the group level, between task based functional responses and RSNs, with all spatial similarity measures being at least 0.83. The results shown in Figure 6 do not include all 13 present RSNs. However, they suggest the possibility that a more complete account of the associations between 3DCNN-mapped RSNs and specific cognitive processes may be feasible. Achieving this objective is of great importance from the clinical and research perspectives.

From the clinical perspective, secure functional localization can serve as the basis for expanding the use of RS-fMRI for pre-surgical applications (17, 21). There are numerous advantages of using RS-fMRI for clinical brain mapping in comparison to task based paradigms. While preoperative fMRI can significantly improve long term survival in the setting of surgical resection of malignant brain tumors, its dependence on patient participation limits who can access the mapping (i.e., children and modestly impaired patients) (77, 78). The need for personnel to administer the scans and the long duration of acquisition limits how often the task-based techniques are available. Also, when clinically applied for preoperative brain mapping task-based fMRI has a high failure rate. The limitations of current mapping methods are obviated by the use of RS-fMRI generally. RS-fMRI is robust, highly efficient, requires minimal task compliance, and has a substantially reduced failure rate when used clinically (when compared to task-based methods). Our method has the potential to further simplify and streamline brain mapping by automating functional localization and limiting the need for significant technical and scientific expertise that are required with more classic approaches (e.g,. seed-based mapping).

A number of limitations and future directions exist in relation to the present study. The current study primarily focused on results in the context of group averages. While we have provided STD maps which reflect voxelwise variability between subjects, future work should focus on a more detailed analysis of individual subject variability between probability maps produced by the 3DCNN. Similarly, differences in networks due to demographics, such as age and gender should be investigated. Second, while there was a high degree of similarity between our results and other published resting state and task network maps, there were some differences. Future work could involve an in-depth comparison of the topographical differences observed among the various published network maps. Lastly, our model showed little difference when comparing results derived using 8,000 time points (~300 min) compared to results from ~150 time points (~5 min). While these results are promising, future work should focus specifically on models optimized for producing probability maps based on data sets with fewer BOLD time points. This could include investigating different network architectures and hyperparameters and their impact on classification accuracy, as well as identifying the optimal number of samples required for the given network.

### 4.1. Conclusion

In this work, we have demonstrated the utility of deep learning for accurate probabilistic mapping of resting state networks in the brain. This method is robust to noisy and small data sets. We demonstrate the similarity between our results and other previously published task and resting state segmentations. RSN probability maps are made publicly available, and maybe helpful for future studies interested in ROIs computed from a large data set (>2,000) of normal adults.

## Data availability statement

The original contributions presented in the study (mean and STD probability maps) are included in the article/Supplementary material, further inquiries can be directed to the corresponding author.

## Ethics statement

The studies involving human participants were reviewed and approved by Washington University Institutional Review Board. The patients/participants provided their written informed consent to participate in this study.

## Author contributions

PL: conceptualization, methodology, software, validation, formal analysis, investigation, data curation, writing—original draft, and visualization. JL: methodology, software, formal analysis, data curation, writing—original draft, and visualization. KP and RR: software, resources, data curation, writing—original draft, and visualization. KM: conceptualization. EG: methodology and writing—original draft. AS: software, writing—original draft, and supervision. BA: resources, writing—original draft, supervision, project administration, and funding acquisition. EL: conceptualization, resources, writing—original draft, supervision, project administration, and funding acquisition. JS: conceptualization, methodology, writing—original draft, supervision, project administration, and funding acquisition. All authors contributed to the article and approved the submitted version.

## References

[1] Biswal B Zerrin Yetkin F Haughton VM Hyde JS. Functional connectivity in the motor cortex of resting human brain using echo-planar MRI. Magn Reson Med. (1995) 34:537–41. 10.1002/mrm.1910340409 8524021

[2] Beckmann CF DeLuca M Devlin JT Smith SM. Investigations into resting-state connectivity using independent component analysis. Philos Trans R Soc B Biol Sci. (2005) 360:1001–13. 10.1098/rstb.2005.1634 16087444 PMC1854918

[3] Seeley WW Menon V Schatzberg AF Keller J Glover GH Kenna H. Dissociable intrinsic connectivity networks for salience processing and executive control. J Neurosci. (2007) 27:2349–56. 10.1523/JNEUROSCI.5587-06.2007 17329432 PMC2680293

[4] Buzsáki G. Large-scale recording of neuronal ensembles. Nat Neurosci. (2004) 7:446–51. 10.1038/nn1233 15114356

[5] De Pasquale F Della Penna S Snyder AZ Marzetti L Pizzella V Romani GL. cortical core for dynamic integration of functional networks in the resting human brain. Neuron. (2012) 74:753–64. 10.1016/j.neuron.2012.03.031 22632732 PMC3361697

[6] Fox MD Snyder AZ Vincent JL Corbetta M Van Essen DC Raichle ME. The human brain is intrinsically organized into dynamic, anticorrelated functional networks. Proc Natl Acad Sci U S A. (2005) 3:6102. 10.1073/pnas.0504136102 15976020 PMC1157105

[7] Fox MD Raichle ME. Spontaneous fluctuations in brain activity observed with functional magnetic resonance imaging. Nat Rev Neurosci. (2007) 8:700–11. 10.1038/nrn2201 17704812

[8] Paul RH Cho KS Luckett PM Strain JF Belden AC Bolzenius JD. Machine learning analysis reveals novel neuroimaging and clinical signatures of frailty in HIV. JAIDS J Acquir Immune Defic Syndr. (2020) 3:2360. 10.1097/QAI.0000000000002360 32251142 PMC7903919

[9] Khosla M Jamison K Ngo GH Kuceyeski A Sabuncu MR. Machine learning in resting-state fMRI analysis. Magn Reson Imaging. (2019) 64:101–21. 10.1016/j.mri.2019.05.031 31173849 PMC6875692

[10] Strain JF Brier MR Tanenbaum A Gordon BA McCarthy JE Dincer A. Covariance-based vs. correlation-based functional connectivity dissociates healthy aging from Alzheimer disease. Neuroimage. (2022) 261:119511. 10.1016/j.neuroimage.2022.119511 35914670 PMC9750733

[11] Smith RX Strain JF Tanenbaum A Fagan AM Hassenstab J McDade E. Resting-state functional connectivity disruption as a pathological biomarker in autosomal dominant Alzheimer disease. Brain Connect. (2021) 11:239–49. 10.1089/brain.2020.0808 33430685 PMC8182476

[12] Campbell MC Koller JM Snyder AZ Buddhala C Kotzbauer PT Perlmutter JS. proteins and resting-state functional connectivity in Parkinson disease. Neurology. (2015) 84:2413–21. 10.1212/WNL.0000000000001681 25979701 PMC4478033

[13] Anderson JS Nielsen JA Ferguson MA Burback MC Cox ET Dai L Gerig G Korenberg JR. Abnormal brain synchrony in Down Syndrome. NeuroImage Clin. (2013) 10.1016/j.nicl.2013.05.006 24179822 PMC3778249

[14] Rausch A Zhang W Haak KV Mennes M Hermans EJ Van Oort E. Altered functional connectivity of the amygdaloid input nuclei in adolescents and young adults with autism spectrum disorder: A resting state fMRI study. Mol Autism. (2016) 5:60. 10.1186/s13229-015-0060-x 26823966 PMC4730628

[15] Gulati S Jakola AS Nerland US Weber C Solheim O. The risk of getting worse: surgically acquired deficits, perioperative complications, and functional outcomes after primary resection of glioblastoma. World Neurosurg. (2011) 76:572–9. 10.1016/j.wneu.2011.06.014 22251506

[16] Fox MD Halko MA Eldaief MC Pascual-Leone A. Measuring and manipulating brain connectivity with resting state functional connectivity magnetic resonance imaging. (fcMRI) and transcranial magnetic stimulation (TMS). Neuroimage. (2012) 62:2232–43. 10.1016/j.neuroimage.2012.03.035 22465297 PMC3518426

[17] Hacker CD Roland JL Kim AH Shimony JS Leuthardt EC. Resting-state network mapping in neurosurgical practice: s review. Neurosurg Focus. (2019) 3.9656. 10.3171/2019.9.FOCUS19656 31786561 PMC9841914

[18] Sair HI Agarwal S Pillai JJ. Application of resting state functional MR imaging to presurgical mapping: Language mapping. Neuroimaging Clin. (2017) 27:635–44. 10.1016/j.nic.2017.06.003 28985934

[19] Catalino MP Yao S Green DL Laws ER Golby AJ Tie Y. Mapping cognitive and emotional networks in neurosurgical patients using resting-state functional magnetic resonance imaging. Neurosurg Focus. (2020) 48:E9. 10.3171/2019.11.FOCUS19773 32006946 PMC7712886

[20] Rosazza C Aquino D D'Incerti L Cordella R Andronache A Zacà D. Preoperative mapping of the sensorimotor cortex: comparative assessment of task-based and resting-state FMRI. PLoS One. (2014) 9:e98860. 10.1371/journal.pone.0098860 24914775 PMC4051640

[21] Leuthardt EC Guzman G Bandt SK Hacker C Vellimana AK Limbrick D. Integration of resting state functional MRI into clinical practice-A large single institution experience. PLoS ONE. (2018) 13:e0198349. 10.1371/journal.pone.0198349 29933375 PMC6014724

[22] Miller KL Alfaro-Almagro F Bangerter NK Thomas DL Yacoub E Xu J. Multimodal population brain imaging in the UK Biobank prospective epidemiological study. Nat Neurosci. (2016) 19:1523–36. 10.1038/nn.4393 27643430 PMC5086094

[23] Alom MZ Taha TM Yakopcic C Westberg S Sidike P Nasrin MS. state-of-the-art survey on deep learning theory and architectures. Electronics. (2019) 8:292. 10.3390/electronics8030292

[24] Mazurowski MA Buda M Saha A Bashir MR. Deep learning in radiology: An overview of the concepts and a survey of the state of the art with focus on MRI. J Magn Reson imaging. (2019) 49:939–54. 10.1002/jmri.26534 30575178 PMC6483404

[25] Akkus Z Galimzianova A Hoogi A Rubin DL Erickson BJ. Deep learning for brain MRI segmentation: state of the art and future directions. J Digit Imaging. (2017) 5:9983. 10.1007/s10278-017-9983-4 28577131 PMC5537095

[26] Yala A Lehman C Schuster T Portnoi T Barzilay R. A deep learning mammography-based model for improved breast cancer risk prediction. Radiology. (2019) 292:60–6. 10.1148/radiol.2019182716 31063083

[27] Ahmed Z Mohamed K Zeeshan S Dong X. Artificial intelligence with multi-functional machine learning platform development for better healthcare and precision medicine. Database. (2020) 2020:10. 10.1093/database/baaa010 32185396 PMC7078068

[28] Zhang S Bamakan SMH Qu Q Li S. Learning for personalized medicine: a comprehensive review from a deep learning perspective. IEEE Rev Biomed Eng. (2018) 12:194–208. 10.1109/RBME.2018.2864254 30106692

[29] MacEachern SJ Forkert ND. Machine learning for precision medicine. Genome. (2021) 64:416–25. 10.1139/gen-2020-0131 33091314

[30] Zhu G Jiang B Tong L Xie Y Zaharchuk G Wintermark M. Applications of deep learning to neuro-imaging techniques. Front Neurol. (2019) 12:869. 10.3389/fneur.2019.00869 31474928 PMC6702308

[31] Gong E Pauly JM Wintermark M Zaharchuk G. Deep learning enables reduced gadolinium dose for contrast-enhanced brain MRI. J Magn Reson imaging. (2018) 48:330–40. 10.1002/jmri.25970 29437269

[32] Xie S Zheng X Chen Y Xie L Liu J Zhang Y. Artifact removal using improved GoogLeNet for sparse-view CT reconstruction. Sci Rep. (2018) 8:1–9. 10.1038/s41598-018-25153-w 29712978 PMC5928081

[33] Gupta H Jin KH Nguyen HQ McCann MT Unser M. CNN-based projected gradient descent for consistent CT image reconstruction. IEEE Trans Med Imaging. (2018) 37:1440–53. 10.1109/TMI.2018.2832656 29870372

[34] Kaplan S Zhu Y-M. Full-dose PET image estimation from low-dose PET image using deep learning: a pilot study. J Digit Imaging. (2019) 32:773–8. 10.1007/s10278-018-0150-3 30402670 PMC6737135

[35] Golkov V Dosovitskiy A Sperl JI Menzel MI Czisch M Sämann P. Q-space deep learning: twelve-fold shorter and model-free diffusion MRI scans. IEEE Trans Med Imaging. (2016) 35:1344–51. 10.1109/TMI.2016.2551324 27071165

[36] Zhu B Liu JZ Cauley SF Rosen BR Rosen MS. Image reconstruction by domain-transform manifold learning. Nature. (2018) 555:487–92. 10.1038/nature25988 29565357

[37] Dworetsky A Seitzman BA Adeyemo B Neta M Coalson RS Petersen SE. Probabilistic mapping of human functional brain networks identifies regions of high group consensus. Neuroimage. (2021) 237:118164. 10.1016/j.neuroimage.2021.118164 34000397 PMC8296467

[38] Yarkoni T Poldrack RA Nichols TE Van Essen DC Wager TD. Large-scale automated synthesis of human functional neuroimaging data. Nat Methods. (2011) 8:665–70. 10.1038/nmeth.1635 21706013 PMC3146590

[39] Gordon EM Laumann TO Gilmore AW Newbold DJ Greene DJ Berg JJ. Precision functional mapping of individual human brains. Neuron. (2017) 95:791–807. 10.1016/j.neuron.2017.07.011 28757305 PMC5576360

[40] Buckner RL Roffman JL Smoller JW. Brain Genomics Superstruct Project. (GSP). Harvard Dataverse (2014).

[41] Power JD Mitra A Laumann TO Snyder AZ Schlaggar BL Petersen SE. Methods to detect, characterize, and remove motion artifact in resting state fMRI. Neuroimage. (2014) 45:48. 10.1016/j.neuroimage.2013.08.048 23994314 PMC3849338

[42] Hacker CD Laumann TO Szrama NP Baldassarre A Snyder AZ Leuthardt EC Corbetta M. Resting state network estimation in individual subjects. Neuroimage. (2013) 20:108. 10.1016/j.neuroimage.2013.05.108 23735260 PMC3909699

[43] Smyser CD Inder TE Shimony JS Hill JE Degnan AJ Snyder AZ Neil JJ. Longitudinal analysis of neural network development in preterm infants. Cereb Cortex. (2010) 35:35. 10.1093/cercor/bhq035 20237243 PMC2978240

[44] Fox MD Zhang D Snyder AZ Raichle ME. The global signal and observed anticorrelated resting state brain networks. J Neurophysiol. (2009) 101:3270–83. 10.1152/jn.90777.2008 19339462 PMC2694109

[45] Huang G Liu Z Van Der Maaten L Weinberger KQ. Densely connected convolutional networks. Proceedings - 30th IEEE Conference on Computer Vision and Pattern Recognition, CVPR 2017. (2017). 10.1109/CVPR.2017.243

[46] He K Zhang X Ren S Sun J. Deep residual learning for image recognition. Proceedings of the IEEE Computer Society Conference on Computer Vision and Pattern Recognition. Las Vegas, NV. (2016). 10.1109/CVPR.2016.9032166560

[47] Lin M Chen Q Yan S. Network in network. 2nd International Conference on Learning Representations, ICLR 2014 - Conference Track Proceedings. Banff, AB. (2014).

[48] Wu H Gu X. Towards dropout training for convolutional neural networks. Neural Networks. (2015) 3:7. 10.1016/j.neunet.2015.07.007 26277608

[49] Yu D Wang H Chen P Wei Z. Mixed pooling for convolutional neural networks. Lect Notes Comp Sci. (2014) 45:34. 10.1007/978-3-319-11740-9_34

[50] Seitzman BA Gratton C Marek S Raut R V Dosenbach NUF Schlaggar BL. set of functionally-defined brain regions with improved representation of the subcortex and cerebellum. Neuroimage. (2020) 206:116290. 10.1016/j.neuroimage.2019.116290 31634545 PMC6981071

[51] Shorten C Khoshgoftaar TM. A survey on image data augmentation for deep learning. J Big Data. (2019) 21:197. 10.1186/s40537-019-0197-0 PMC8287113 34306963

[52] Gordon EM Laumann TO Marek S Newbold DJ Hampton JM Seider NA. Individualized functional subnetworks connect human striatum and frontal cortex. Cereb Cortex. (2021) 15:387. 10.1093/cercor/bhab387 34718460 PMC9247416

[53] Raut R V Mitra A Marek S Ortega M Snyder AZ Tanenbaum A. Organization of propagated intrinsic brain activity in individual humans. Cereb Cortex. (2020) 30:1716–34. 10.1093/cercor/bhz198 31504262 PMC7132930

[54] Laumann TO Snyder AZ Mitra A Gordon EM Gratton C Adeyemo B. On the stability of BOLD fMRI correlations. Cereb cortex. (2017) 27:4719–32. 10.1093/cercor/bhw265 27591147 PMC6248456

[55] Wang Z Simoncelli EP Bovik AC. Multiscale structural similarity for image quality assessment. The Thrity-Seventh Asilomar Conference on Signals, Systems & Computers, 2003. Ieee. (2003). p. 1398–1402

[56] Wang Z Bovik AC Sheikh HR Simoncelli EP. Image quality assessment: from error visibility to structural similarity. IEEE Trans image Process. (2004) 13:600–12. 10.1109/TIP.2003.819861 15376593

[57] Van Essen DC Glasser MF Dierker DL Harwell J Coalson T. Parcellations and hemispheric asymmetries of human cerebral cortex analyzed on surface-based atlases. Cereb cortex. (2012) 22:2241–62. 10.1093/cercor/bhr291 22047963 PMC3432236

[58] Noble S. Reliability and Validity of fMRI Mapping Methods. Ann Arbor, MI: ProQuest (2019).

[59] Kumar V Minz S. Feature selection: a literature review. SmartCR. (2014) 4:211–29. 10.6029/smartcr.2014.03.007

[60] Doucet G Naveau M Petit L Delcroix N Zago L Crivello F. Brain activity at rest: a multiscale hierarchical functional organization. J Neurophysiol. (2011) 105:2753–63. 10.1152/jn.00895.2010 21430278

[61] Gotts SJ Gilmore AW Martin A. Brain networks, dimensionality, and global signal averaging in resting-state fMRI: Hierarchical network structure results in low-dimensional spatiotemporal dynamics. Neuroimage. (2020) 205:116289. 10.1016/j.neuroimage.2019.116289 31629827 PMC6919311

[62] Liu X Ward BD Binder JR Li SJ Hudetz AG. Scale-free functional connectivity of the brain is maintained in anesthetized healthy participants but not in patients with unresponsive wakefulness syndrome. PLoS ONE. (2014) 9:e92182. 10.1371/journal.pone.0092182 24647227 PMC3960221

[63] Yeo BTT Krienen FM Sepulcre J Sabuncu MR Lashkari D Hollinshead M. The organization of the human cerebral cortex estimated by intrinsic functional connectivity. J Neurophysiol. (2011) 106:1125–65. 10.1152/jn.00338.2011 21653723 PMC3174820

[64] Lee MH Hacker CD Snyder AZ Corbetta M Zhang D Leuthardt EC. Clustering of resting state networks. PLoS ONE. (2012) 7:e40370. 10.1371/journal.pone.0040370 22792291 PMC3392237

[65] Buckner RL Krienen FM Castellanos A Diaz JC Thomas Yeo BT. The organization of the human cerebellum estimated by intrinsic functional connectivity. J Neurophysiol. (2011) 35:2011. 10.1152/jn.00339.2011 21795627 PMC3214121

[66] Power JD Cohen AL Nelson SM Wig GS Barnes KA Church JA. Functional network organization of the human brain. Neuron. (2011) 72:665–78. 10.1016/j.neuron.2011.09.006 22099467 PMC3222858

[67] Ji JL Spronk M Kulkarni K Repovš G Anticevic A Cole MW. Mapping the human brain's cortical-subcortical functional network organization. Neuroimage. (2019) 185:35–57. 10.1016/j.neuroimage.2018.10.006 30291974 PMC6289683

[68] Marek S Siegel JS Gordon EM Raut R V Gratton C Newbold DJ. Spatial and temporal organization of the individual human cerebellum. Neuron. (2018) 100:977–93. 10.1016/j.neuron.2018.10.010 30473014 PMC6351081

[69] Glasser MF Coalson TS Robinson EC Hacker CD Harwell J Yacoub E. multi-modal parcellation of human cerebral cortex. Nature. (2016) 536:171–8. 10.1038/nature18933 27437579 PMC4990127

[70] Schaefer A Kong R Gordon EM Laumann TO Zuo X-N Holmes AJ. Local-global parcellation of the human cerebral cortex from intrinsic functional connectivity MRI. Cereb cortex. (2018) 28:3095–114. 10.1093/cercor/bhx179 28981612 PMC6095216

[71] Buckner RL Krienen FM. The evolution of distributed association networks in the human brain. Trends Cogn Sci. (2013) 17:648–65. 10.1016/j.tics.2013.09.017 24210963

[72] Laumann TO Gordon EM Adeyemo B Snyder AZ Joo SJ Chen M-Y. Functional system and areal organization of a highly sampled individual human brain. Neuron. (2015) 87:657–70. 10.1016/j.neuron.2015.06.037 26212711 PMC4642864

[73] Bijsterbosch J Harrison S Duff E Alfaro-Almagro F Woolrich M Smith S. Investigations into within-and between-subject resting-state amplitude variations. Neuroimage. (2017) 159:57–69. 10.1016/j.neuroimage.2017.07.014 28712995 PMC5678294

[74] Poldrack RA Yarkoni T. From brain maps to cognitive ontologies: informatics and the search for mental structure. Annu Rev Psychol. (2016) 67:587. 10.1146/annurev-psych-122414-033729 26393866 PMC4701616

[75] Mineroff Z Blank IA Mahowald K Fedorenko E A. robust dissociation among the language, multiple demand, and default mode networks: Evidence from inter-region correlations in effect size. Neuropsychologia. (2018) 119:501–11. 10.1016/j.neuropsychologia.2018.09.011 30243926 PMC6191329

[76] Bertolero MA Yeo BTT D'Esposito M. The modular and integrative functional architecture of the human brain. Proc Natl Acad Sci. (2015) 112:E6798–807. 10.1073/pnas.1510619112 26598686 PMC4679040

[77] Tan AC Ashley DM López GY Malinzak M Friedman HS Khasraw M. Management of glioblastoma: State of the art and future directions. CA Cancer J Clin. (2020) 70:299–312. 10.3322/caac.21613 32478924

[78] Vysotski S Madura C Swan B Holdsworth R Lin Y Del Rio AM. Preoperative FMRI associated with decreased mortality and morbidity in brain tumor patients. Interdiscip Neurosurg. (2018) 13:40–5. 10.1016/j.inat.2018.02.001 31341789 PMC6653633

