# Supplementary material for: Resting state network mapping in individuals using deep learning
Source: Front Neurol. 2023 Jan 12;13:1055437. doi: 10.3389/fneur.2022.1055437 (PMC9878609; doi:10.3389/fneur.2022.1055437)
Supplement: Supplementary file 1 [file Data_Sheet_1.DOCX]

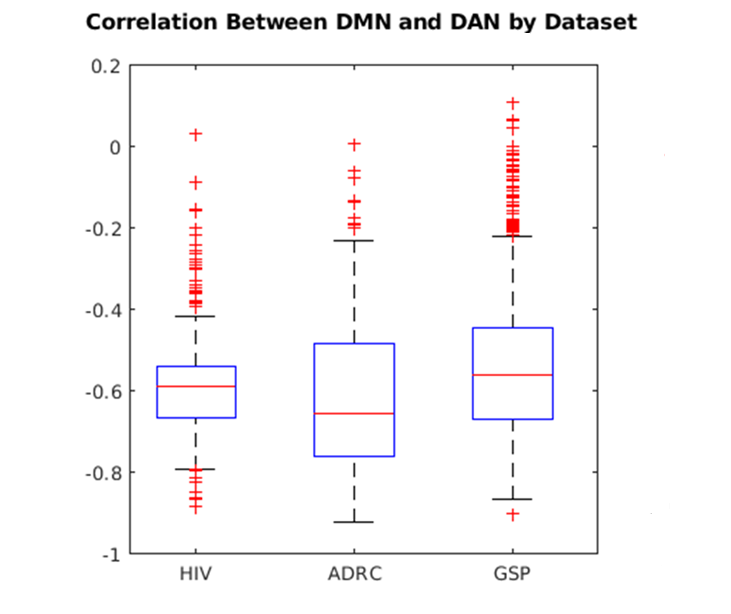


Supplemental Figure 1. Correlations between default mode and dorsal attention networks based on studies used to generate training data. No major differences were observed between studies.


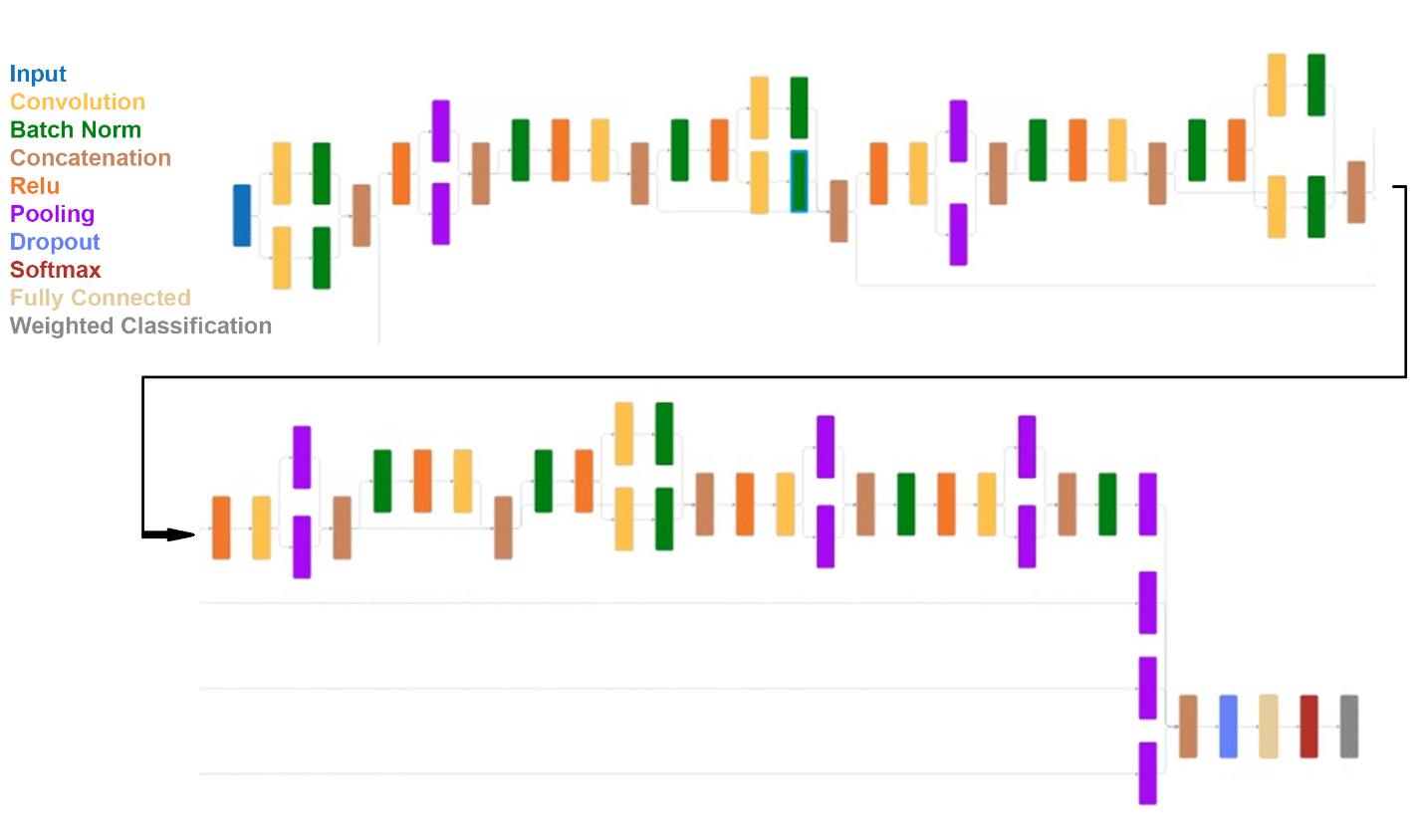


Supplemental Figure 2. 3DCNN architecture.


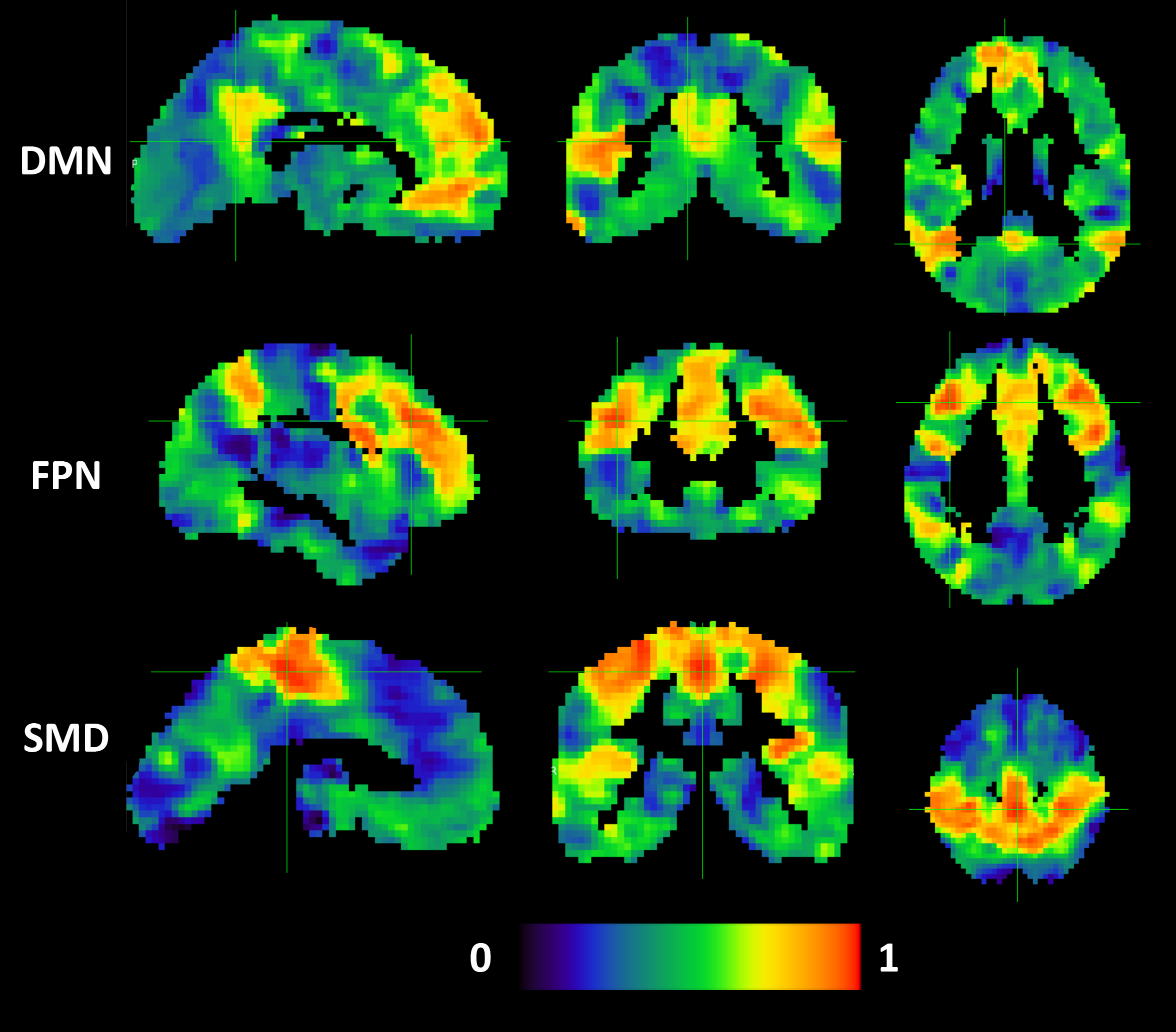


Supplemental Figure 3. Example of training data instance from the default mode (DMN), frontal parietal (FPN), and dorsal somatomotor (SMD) networks.


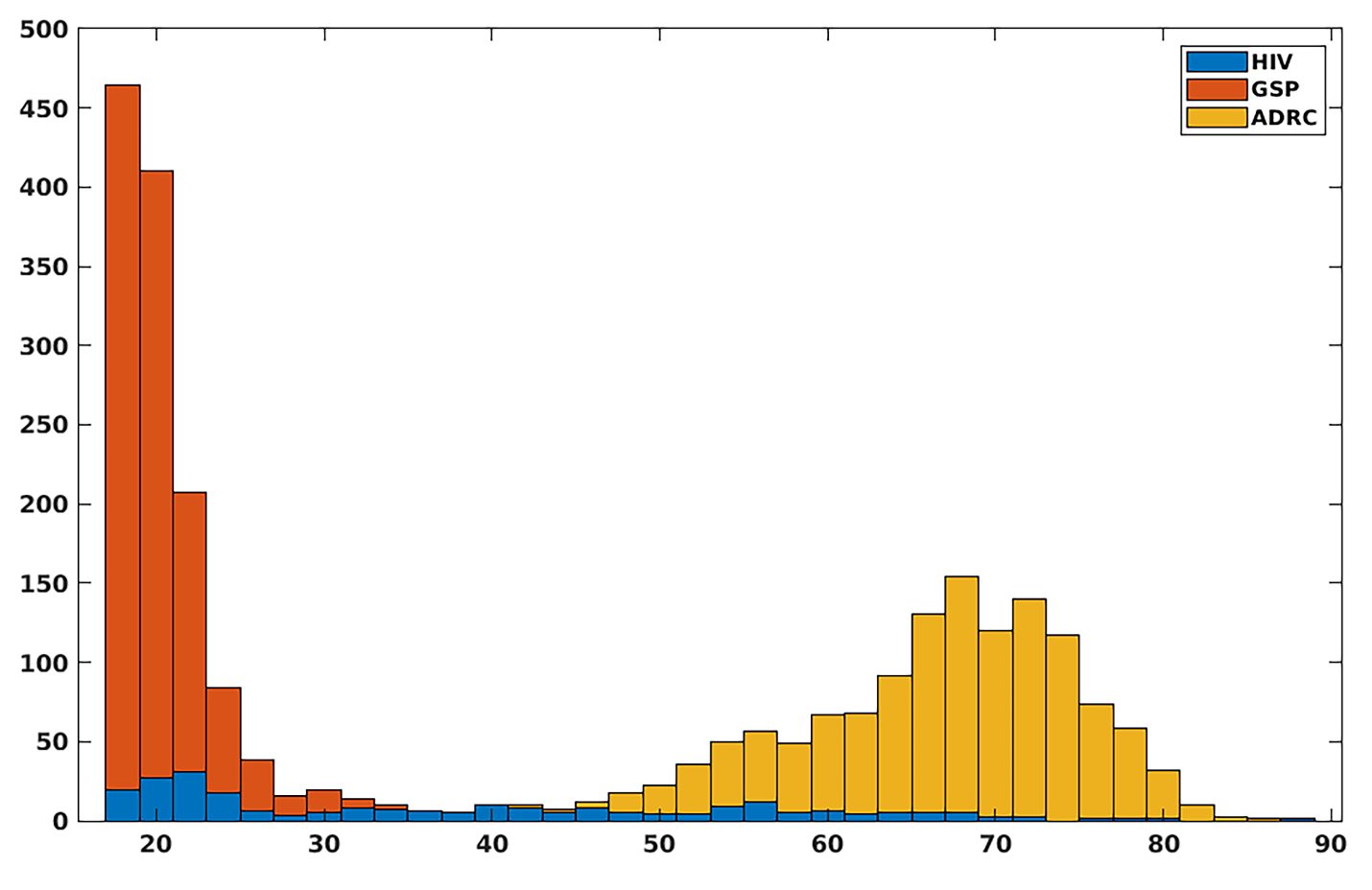


Supplemental Figure 4. Age distribution of data used for training.


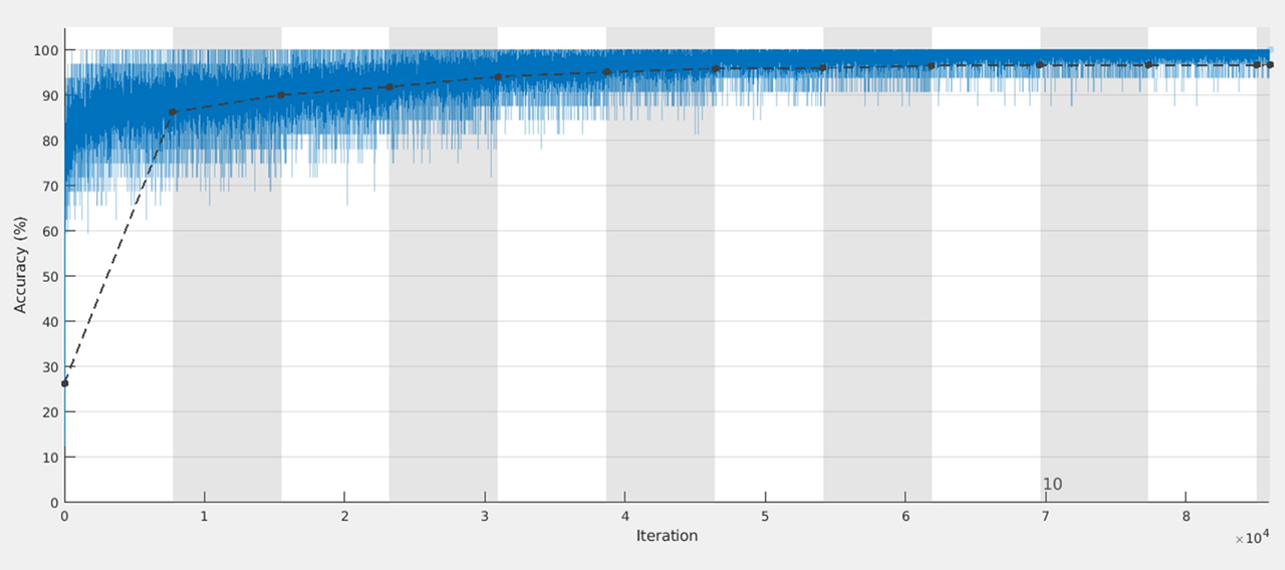


Supplemental Figure 5. Training and validation results for the 3DCNN. The model achieved 99% accuracy on training data (blue) and 97% accuracy on out of sample validation data (black) after 8 epochs.


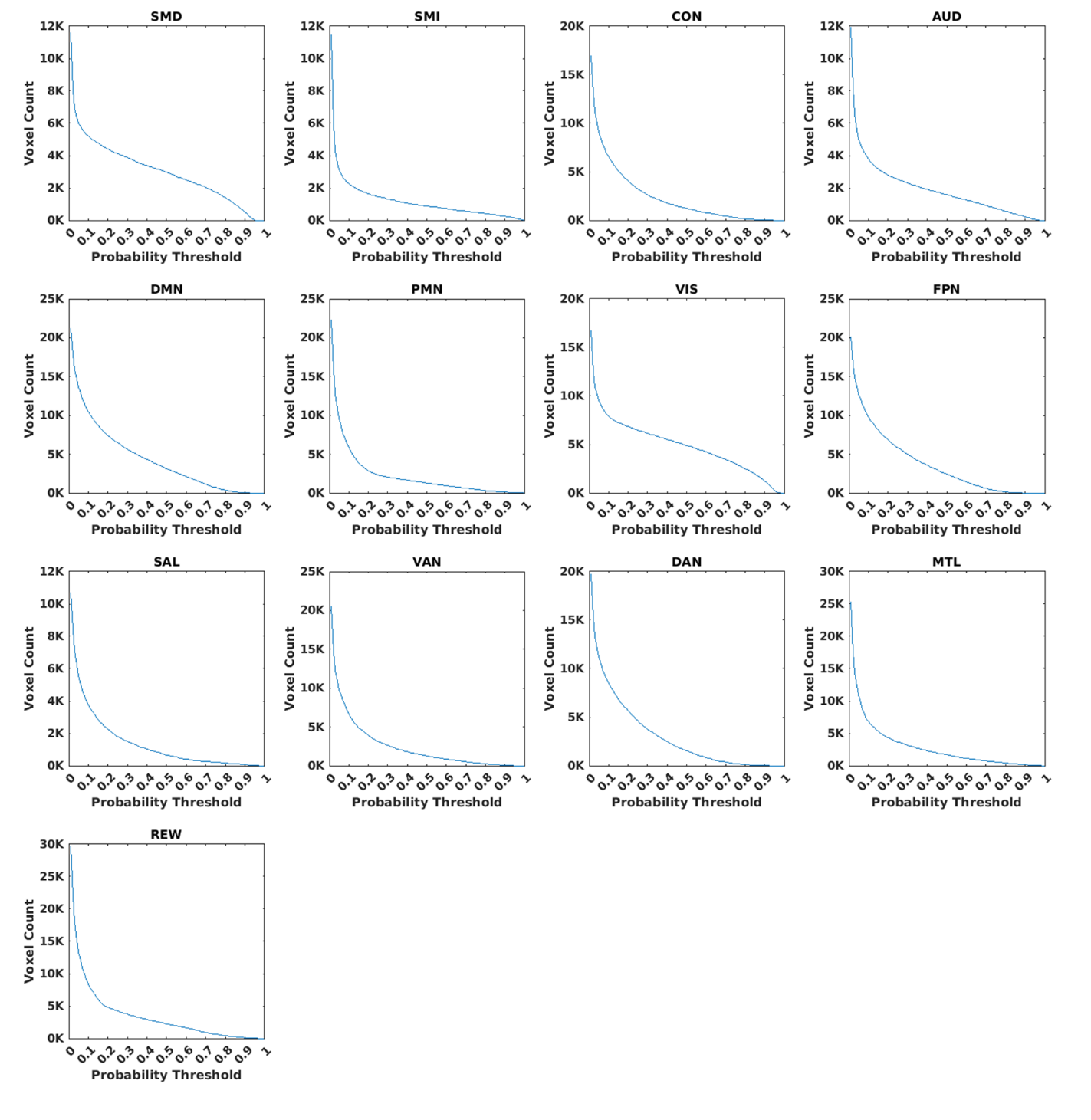


Supplemental Figure 6. Number of voxels per network based on a given probability threshold


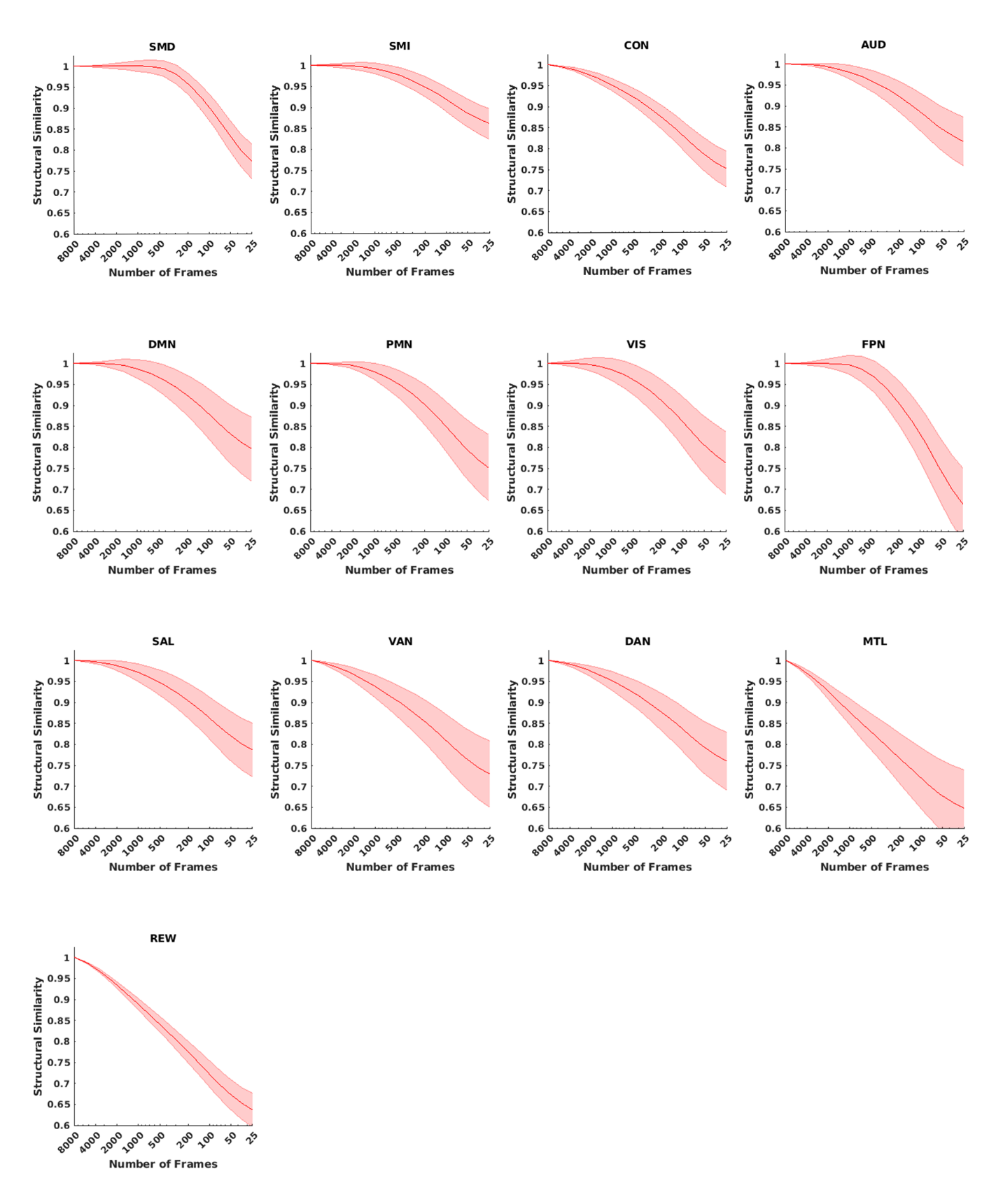


Supplemental Figure 7. Effect of reducing time series for individual networks
